# Supplementary material for: Rebuilding of destroyed spin squeezing in noisy environments
Source: Sci Rep. 2017 Oct 26;7:14102. doi: 10.1038/s41598-017-14442-5 (PMC5658406; doi:10.1038/s41598-017-14442-5)
Supplement: Supplementary file 1 — Supplementary Materials [file 41598_2017_14442_MOESM1_ESM.pdf]

Supplementary Materials : Rebuilding of destroyed spin squeezing in noisy environments

Peng Xu[1], Huanying Sun[1], S. Yi[2], and Wenxian Zhang[1,*]

[1]School of Physics and Technology, Wuhan University, Wuhan, Hubei 430072, China
[2]CAS Key Laboratory of Theoretical Physics, Institute of Theoretical Physics, Chinese Academy of Sciences, P.O. Box 2735, Beijing 100190, China
[*]Corresponding email: wxzhang@whu.edu.cn

ABSTRACT

This is supplementary materials for the paper "Rebuilding of destroyed spin squeezing in noisy environments".

Effect of n_0 on the dynamics of driven-OAT

We have neglected the term with $\hat{n}_0$ from Eq. (2) to Eq. (3) in a zero magnetic field. We justify this approximation by comparing the optimal squeezing dynamics with and without the $\hat{n}_0$ term, as shown in Fig. 1. Clearly, when the number of atoms $N > 10$, the effect of the $\hat{n}_0$ term can be safely neglected.

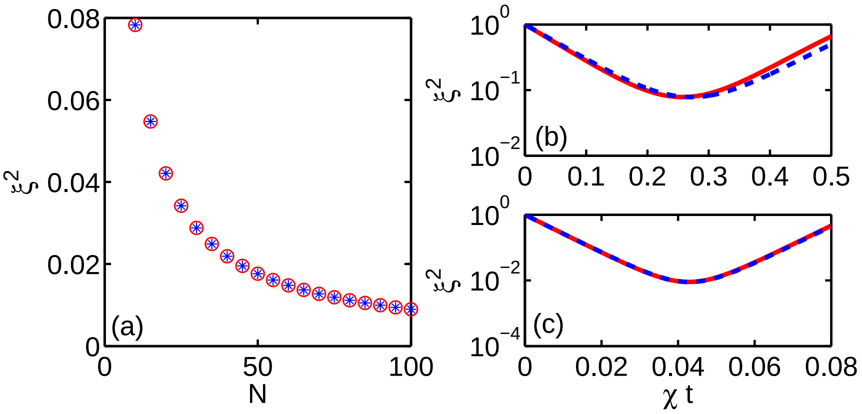

Figure 1. (a) Optimal squeezing parameters for driven-OAT with (blue asterisks) and without (red circles) the $\hat{n}_0$ term. (b) and (c) show the squeezing dynamics with (blue dashed lines) and without (red lines) the $\hat{n}_0$ term for $N = 10$ and 100, respectively. The term with $\hat{n}_0$ shows very little effect on the squeezing dynamics if $N > 10$.

Fisher information during the "two-axis twisting"

In quantum metrology, the sensitivity of a quantum measurement utilizing a squeezed spin state is limited by the quantum Cramer-Rao bound[1–4]

$$\Delta\gamma_Q = \frac{1}{\sqrt{F_Q}}$$

where the quantum Fisher information F_Q for the squeezed spin state is[1,3,5]

$$F_Q = 4(\Delta J_{\alpha+\pi/2})^2.$$

where α is the optimal squeezing direction. Such a quantum limit of the sensitivity is often tested by the Ramsey interferometry[6,7].

We show in Fig. 2 the time dependence of the quantum Fisher information and the sensitivity, respectively, for the ideal driven-OAT. We find that the value of the minimal sensitivity is $\Delta\gamma_Q = 3.13\times10^{-4}$ at $\chi t = 5.80\times10^{-3}$, which is close to that of the optimal spin squeezing parameter.

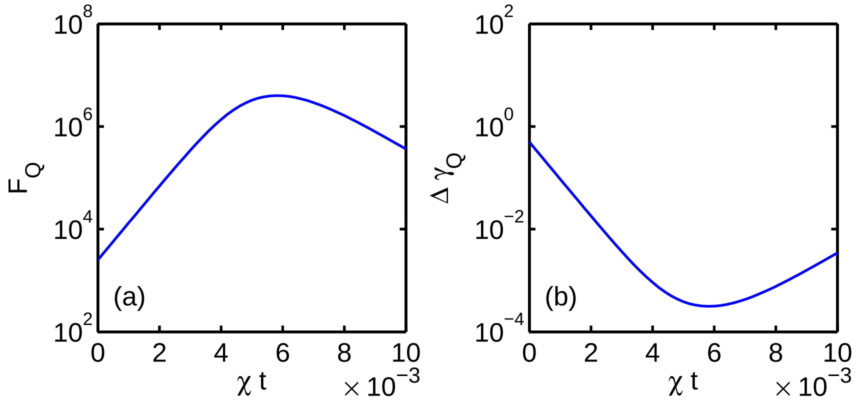

Figure 2. Evolution of (a) quantum Fisher information and (b) phase sensitivity for the ideal driven-OAT (blue solid lines).

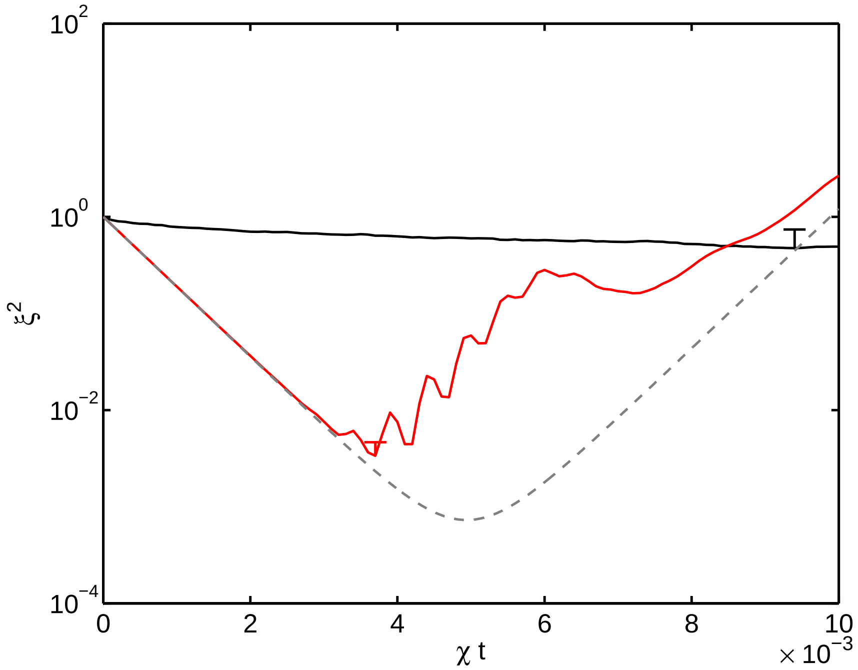

Figure 3. Averaged driven-OAT evolution (over 30 samples) of spin squeezing parameters in stray ac magnetic fields with CDD pulses (red solid curve) and without CDD pulses (black solid curve). $\boldsymbol{\beta}$ is uniformly distributed in a sphere with radius β_m. The grey dashed curve represents the dynamics of TAT results.

Suppression of stray ac magnetic fields

We show in this section that our CDD protocol with a bias field suppresses also the stray ac magnetic fields. As an example, we consider the 50 Hz ac stray magnetic field mainly induced by the electronic devices surrounding the BEC apparatus[8]. Since the frequency and the phase of the ac stray magnetic field can be precisely fixed, we consider only the randomness of the amplitude ($|\boldsymbol{\beta}| \leq \beta_m$ with $\beta_m = 0.1$ mG) and the direction (within 4π solid angle) of the ac fields. We set the bias field as

$h = 50$ mG, the CDD pulse delay $\tau = 490\ \mu$s, and $T_c = 48\tau$. Other parameters about the BEC are the same as in Fig. 3(d) in the main text. Since the simulations of the time dependent stray magnetic fields are very time consuming, we average only 30 runs of the random field amplitudes and directions. However, the statistical error is small enough as indicated by the error bar.

The numerical simulation results are shown in Fig. 3. As comparisons, we plot also the results without CDD pulses and the ideal TAT. Clearly, the optimal spin squeezing parameter with CDD pulses with bias is much smaller than that without CDD pulses. However, the CDD result is above the ideal TAT one, indicating that the developed CDD pulses, aiming at suppressing the static noises, needs to be further improved in order to reach the Heisenberg limit $1/N$.

Deviation for L from ideal TAT results at the vicinity of $\chi t \approx 6 \times 10^{-3}$

In Fig. 3(c) of the main text, the L curve deviates from the ideal TAT results (grey solid line) around $\chi t \approx 6 \times 10^{-3}$. Such a deviation is due to the small effective stray magnetic field $\boldsymbol{\beta}_e$. This field causes the mean spin direction away from z axis, i.e., $\langle J_{x,y} \rangle \neq 0$, around $\chi t \approx 6 \times 10^{-3}$, as shown in Fig. 4(a) and (b) of Supplementary Materials. Note that we use the TAT Hamiltonian in a stray magnetic field, in stead of the driven-OAT. The difference in the squeezing parameter shown in Fig. 4(c) and 4(d) around $\chi t \approx 6 \times 10^{-3}$ implies that the small magnetic field causes the deviation. To understand the physical picture, we present the evolution of the error ellipsoid and the angle φ between the mean spin and the minimal principal axis of the error ellipsoid in Fig. 4(e) and 4(f), respectively. Clearly, the almost coincidence of the circles and the solid lines in Fig. 4(e) indicates that the stray magnetic field changes very little the ellipsoid principal axes (shape) during the evolution. Although the angle φ changes in the order of 10^{-2} in the magnetic field, it causes the squeezing parameter Eq. (6) in the main text soaring up to 1 due to the extremely large long principal axes (in the order of 10^2).

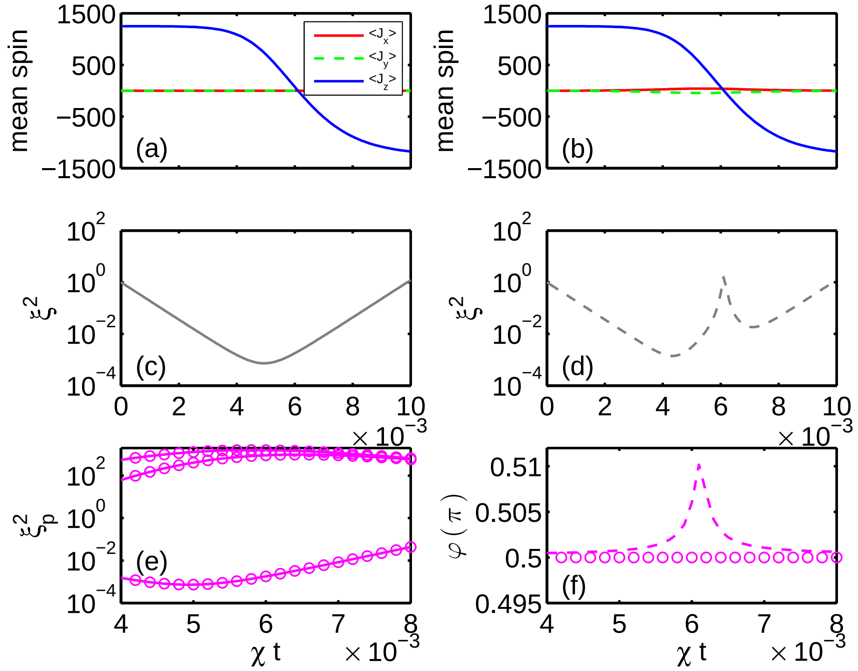

Figure 4. Evolution of the mean spins $\langle J_{x,y,z} \rangle$ for the TAT Hamiltonian in a stray magnetic field $H = H_{TAT} + J_x\beta/3$ with (a) $\beta = 0$ and (b) $\beta = 5\chi$. (c) and (d) are the dynamics of squeezing parameter corresponding to (a) and (b), respectively. (e) Evolution of three principal axes of the error ellipsoid under the Hamiltonian with $\beta = 0$ (purple circles) and $\beta = 5\chi$ (purple solid lines). (f) Evolution of the angles between the mean spin and the minimal principal axis for $\beta = 0$ (purple circles) and $\beta = 5\chi$ (purple dashed line).

References

1. Braunstein, S. L. & Caves, C. M. Statistical distance and the geometry of quantum states. *Phys. Rev. Lett.* **72**, 3439–3443 (1994).

2. Strobel, H. *et al.* Fisher information and entanglement of non-gaussian spin states. *Science* **345**, 424–427 (2014).

3. Pezzé, L. & Smerzi, A. Entanglement, nonlinear dynamics, and the heisenberg limit. *Phys. Rev. Lett.* **102**, 100401 (2009).

4. Hyllus, P., Pezze, L. & Smerzi, A. Entanglement and sensitivity in precision measurements with states of a fluctuating number of particles. *Phys. Rev. Lett.* **105**, 120501 (2010).

5. Wootters, W. K. Statistical distance and hilbert-space. *Phys. Rev. D* **23**, 357–362 (1981).

6. Gross, C., Zibold, T., Nicklas, E., Esteve, J. & Oberthaler, M. K. Nonlinear atom interferometer surpasses classical precision limit. *Nature (London)* **464**, 1165–1169 (2010).

7. Riedel, M. F. *et al.* Atom-chip-based generation of entanglement for quantum metrology. *Nature (London)* **464**, 1170–1173 (2010).

8. Eto, Y. *et al.* Spin-echo-based magnetometry with spinor bose-einstein condensates. *Phys. Rev. A* **88**, 031602 (2013).
